# Cardio-Protective Role of Gingerol along with Prominent Anti-Diabetic Cardiomyopathy Action in A Streptozotocin-Induced Diabetes Mellitus Rat Model

**DOI:** 10.22074/cellj.2017.4509

**Published:** 2017-08-19

**Authors:** Li-Ya Yu, Wen-Lei Shi, Guo Xin-Gui

**Affiliations:** Department of Cardiology, Huadong Hospital, Fudan University, Shanghai, China

**Keywords:** Gingerol, Streptozotocin, Diabetic Cardiomyopathy, Inflammation, Antioxidants

## Abstract

**Objective:**

Diabetic cardiomyopathy (DCM) is characterized as a coronary heart disease which expands during diabetes due to alterations in the myocardial function and structure. The currentstudy intends to elucidate the protective effect of gingerol on DCM in a streptozotocin (STZ)-induced diabetes mellitus (DM) rat model.

**Materials and Methods:**

In this experimental study, the animals were divided into three groups: normal control, DM control, and DM+gingerol (10 mg/kg). The body weights of all rats were estimated at regular intervals. The myocardial profile, oxidative stress, and activities of metabolic enzymes were also scrutinized. The proinflammatory cytokine levels together with cellular protein expression connected with apoptosis were estimated via Western blot analysis.

**Results:**

The rats that suffered from DCM exhibited abnormal levels of myocardial markers, aberrant metabolic enzymatic activity, elevated concentrations of inflammatory factors, and enhanced oxidative stress parameters along with increased cell death apoptosis. Whereas gingerol showed protective effects on the treated rats by an improved antioxidant defense system.

**Conclusion:**

The current findings suggested that gingerol is effective in the treatment of DCM by inhibition of inflammation and oxidative stress.

## Introduction

Diabetes mellitus (DM) arises by numerous etiological factors, most often via abnormal control of lipid and glycometabolism (1). Diabetic cardiomyopathy (DCM) is considered a diabetes complication, which occurs due to changes in myocardial function and structure, systemic hypertension, and considered independent of coronary artery disease. An elevated concentration of free fatty acids and blood lipoproteins can expedite the expansion of cardiovascular disease, including coronary artery disease and hyperlipidemia, which able to escort the additional complications viz., nephropathy, retinopathy, neurosis, hyperglycemia-induced coma, and nephrotoxicity (2,4). The precise mechanism of action of DCM and its etiology are unclear. Oxidative stress plays an imperative role in the expansion of the diabetic complication. Excessive generation of free radicals increases the generation of reactive oxygen species (ROS) and inhibits the mechanism of action of endogenous antioxidant defenses. Inflammatory responses also take part in the expansion of diabetic complications; the inflammatory response speeds up the hyperglycemic conditions for the generation of the delicate response factor of fat cells (5,6). 

Gingerol is known as 5-hydroxy-1-(4-hydroxy- 3-methoxyphenyl) decan-3-one. It is commonly found in fresh ginger and other varieties of piperine and capsicum. Gingerol is a yellow, low-melting crystalline solid. Numerous studies have confirmed its anti-inflammatory, antioxidant and anti-cancer activities, particularly against colon cancer. Most researchers have suggested that gingerol’s play a protective effect in various diseases via an antioxidant mechanism. Several studies reported that free radicals and inflammation process played a crucial role in the expansion of diabetes and its complications. With regards to the well-defined evidence related to the antioxidant and anti-inflammatory effects of gingerol, the present investigation intended to elucidate the protective effects of gingerol against DCM. We sought to confirm the potential benefits of gingerol on DCM and its complications and its involvement in the alteration of cardiac function and linked methods in DM rat models. 

## Materials and Methods

**Fig.1:**
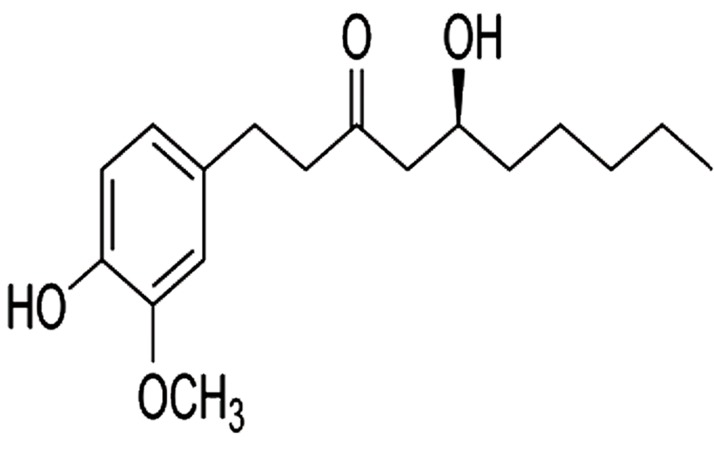
Structure of gingerol.

We purchased gingerol (Fig .1) from Sigma Aldrich (USA). 

### Experimental study

Swiss Albino Wistar rats (80-100 g, male) were used in the current experimental study. The animals were procured form the departmental Animal House and kept in a single cage with excellent ventilation. The rats resided in favorable condition sofa 12-hour light/dark schedule, temperature of 22 ± 5˚C, and relative humidity of 60 ± 5%. The rats received food and water ad libitum before the experimentation. All experimental procedures were performed according to the Instructions for the Care and Use of the Laboratory Animals. The institution’s Ethical Committee approved this experimental study (HHF/16/05). 

### Diabetes induction

The rats received intraperitoneal injections of streptozotocin (STZ) at a dose of 60 mg/kg to induce diabetes. STZ was freshly prepared by dissolving it in a 0.1 M solution of citrate buffer (pH=4.5). The rats fasted overnight before the experiment. Glucose levels of all rats were estimated after 7 days by blood collection from the tail veins. Blood glucose levels of the rats were determined via a glucometer (Johnson and Johnson). Consequently, rats that had blood glucose levels>360 mg/dl were considered to have diabetes (7). 

### Experiment

We divided the rats into the following groups: normal control (group I), STZ-induced diabetic (group II), and STZ-induced diabetic rats that received gingerol (10 mg/kg, group III). Group III rats consumed gingerol dissolved in soybean oil, whereas the normal control and STZ-induced diabetic rats received an equal volume of saline. 

### Estimation of serum myocardial enzymes

We estimated serum myocardial enzymes-aspartate aminotransferase (AST), creatine kinase-MB (CK-MB), and lactate dehydrogenase (LDH). Blood samples from all groups were obtained via the abdominal artery. The collected blood samples were centrifuged at 1500 xg rpm for 15 minutes at 4˚C. Serum myocardial enzymatic activities were estimated according to auto-biochemical methods. 

### Biochemical parameters

We used a glucometer to estimate blood glucose levels. Blood samples from all rats were collected via tail vein puncture. The blood samples were centrifuged at 15000 xg rpm for 15 minutes at 4˚C. The collected supernatant was used to estimate biochemical parameters such as total cholesterol (TC) and serum triglyceride (TG) levels. The biochemical parameters were estimated using an auto analyzer biochemical system (8, 9). The body weight of the all group rats were also estimated at regular intervals. After 2 weeks of gingerol treatment, the rats were euthanized via CO_2_ inhalation.

### Estimation of antioxidant markers

We determined the presence of antioxidant markers from heart tissue. We homogenized the tissue in phosphate buffer (50 mmol/l) at a pH of 7.4. The antioxidant markers, MDA and superoxide dismutase (SOD) were estimated by an earlier reported method (10,11). 

### Western blot determination 

The heart tissues were used to estimate anti- apoptosis proteins BAX, Bcl-2, and caspase-3. The frozen left tissues of the heart sample were mixed with ice-cold lysis buffer, homogenized and centrifuged at 1500 xg for 20 minutes at 4˚C. We used bicinchoninic acid (BCA) to determine protein expressions. 

### Immunohistochemical evaluation

Immunohistochemical analysis was performed on paraffin embedded sections using the microwave dependent antigen retrieval procedure. The processed section was incubated with the standard rabbit polyclonal anti-interlukin-6 (IL-6) and anti-tumor necrosis factor-α (TNF-α) antibodies overnight and constantly treated with biotinylated anti rabbit secondary antibodies for 30 minutes at 37˚C. Aconfocal microscope (A1R+, Nikon) was used for microscopic examinations. 

### Statistical analysis

The results of the current study were reported as the mean ± standard error of the mean. In the current investigation, we performed one-way analysis of variance for statistical analysis (Graphpad Prism). P<0.05 was considered statically significant. 

## Results

### Gingerol prevents metabolic deformity

**Fig.2:**
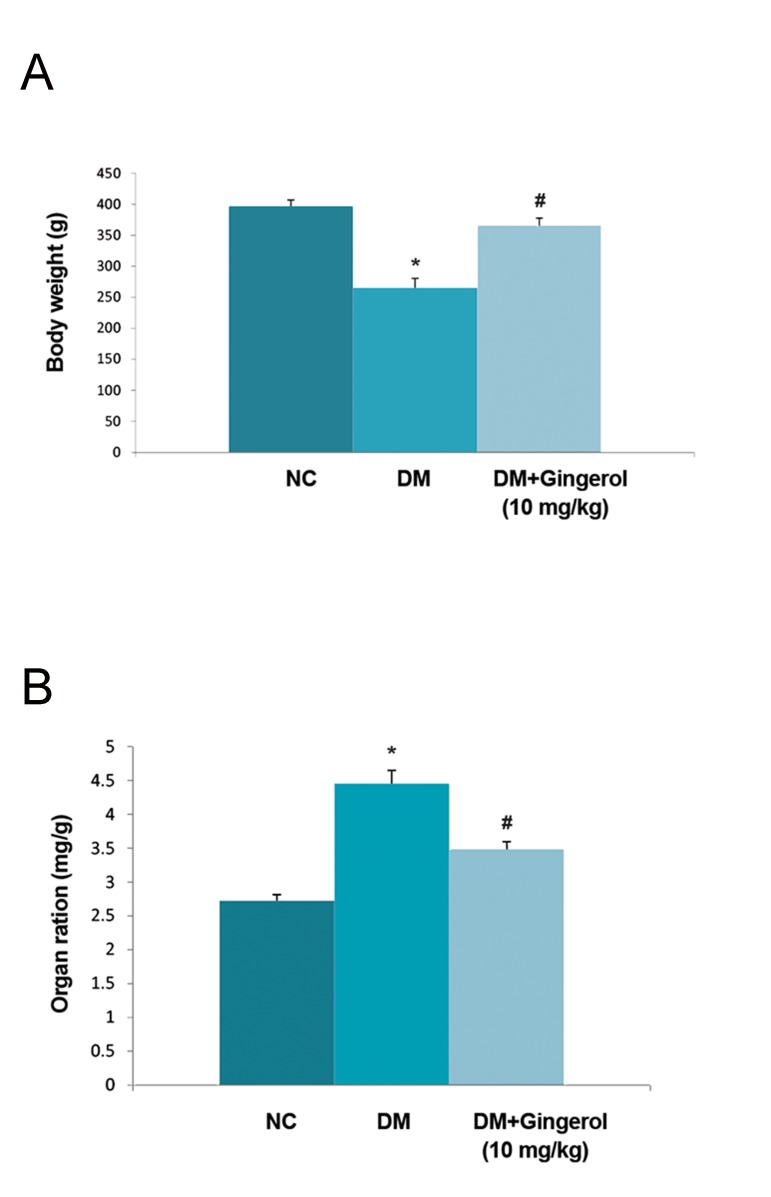
Effect of gingerol on the heart to body weight ratio. A. Body weight variance and B. Heart weight of all the study rats. Data are presented as mean ± standard error of the mean. NC; Normal control, *; P<0.05 vs. control group and #; P<0.05 vs. diabetes mellitus (DM) group.

**Fig.3:**
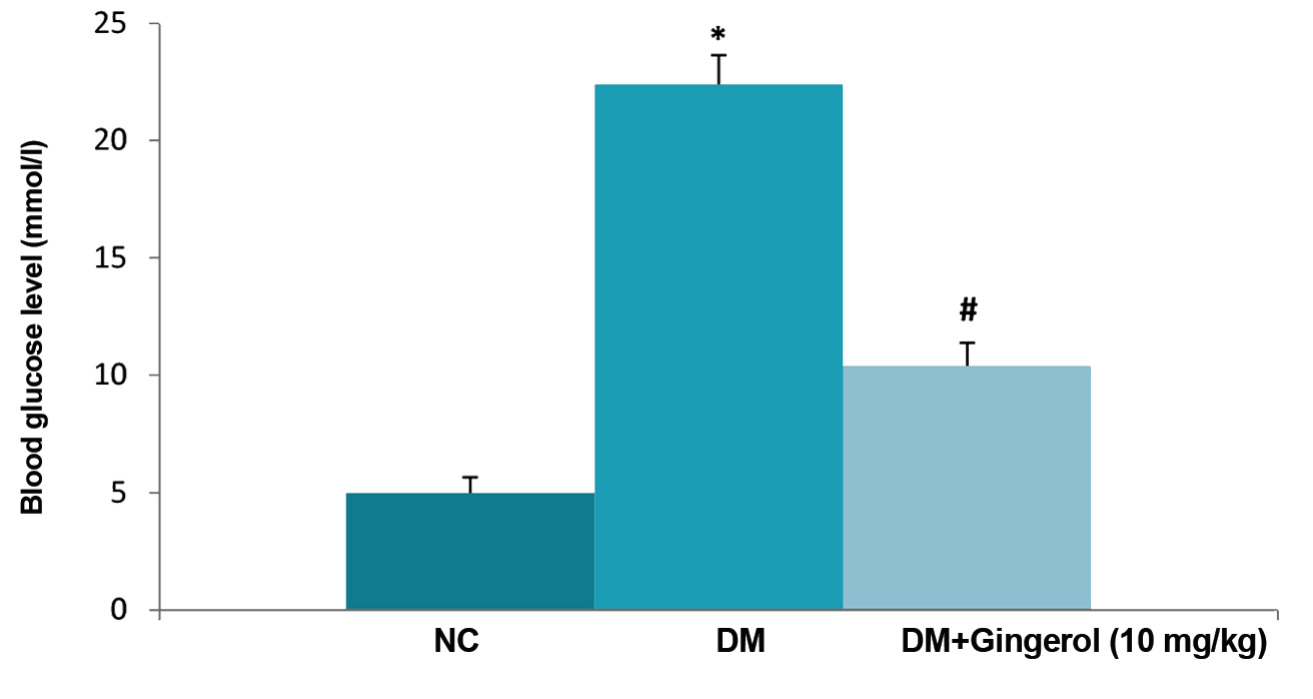
Effect of gingerol on the blood glucose level of rats. The data are presented as mean ± standard error of the mean. NC; Normal control, *; P<0.05 vs. control group and #; P<0.05 vs. diabetes mellitus (DM) group.

**Fig.4:**
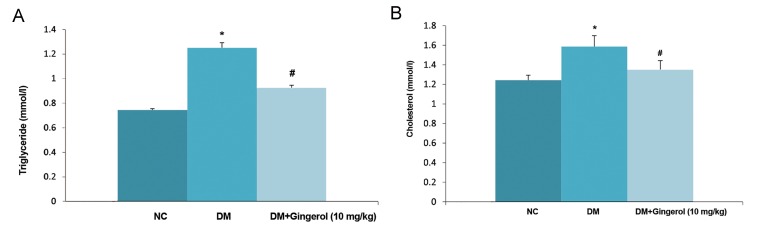
Effect of gingerol on the lipid profile of rats. A. Triglycerides (TG) and B. Total cholesterol (TC) for all the rats. The data are presented as mean ± standard error of the mean. NC; Normal control, *; P<0.05 vs. control group and #; P<0.05 vs. diabetes mellitus (DM) group.

Figure 2A shows the body weights of the normal control, gingerol and STZ-induced DM rats. Normal control group rats had increased body weights compared to STZ-induced DM rats. STZ-induced DM rats had reduced body weights with increased blood glucose levels compared with normal control (NC) group rats. We observed increased body weight ratio in the STZ-induced DM group compared with the NC and gingerol treated groups (Fig .2B). The STZ-induced DM rats that received gingerol had reduced blood glucose levels which approximated the NC group (Fig .3). The STZ- induced DM rats exhibited higher levels of the lipid parameters, TG and TC, compared to the NC group (Fig .4). However, the STZ-induced DM rats treated with gingerol had a significant decrease in TG and TC levels compared to the STZ-induced DM group. 

**Fig.5:**
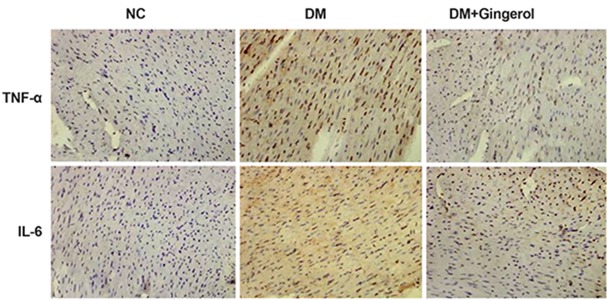
The immunohistochemical staining for myocardial tumor necrosis factor-alpha (TNF-α) and interleukin (IL)-1β expressions. Brown staining indicates cells with positive expression. NC; Normal control and DM; Diabetes mellitus.

### Effect of gingerol on the inflammatory factors

We performed immunohistochemical analysis to estimate the effects of gingerol on the inflammatory mediators, IL-6 and TNF-α. The immunohistochemical study results showed enhanced staining in the STZ-induced DM rats compared to the NC group, which correlated well with the increased level of inflammatory mediators. However, there were decreased inflammatory cytokine levels in the gingerol treated group compared to the STZ-induced DM group. These results suggested a potential effect of gingerol against inflammation (Fig .5). 

### Effect of gingerol on oxidative stress and myocardial damage

Gingerol showed a protective effect against oxidative stress in STZ-induced DM rats. 

**Fig.6:**
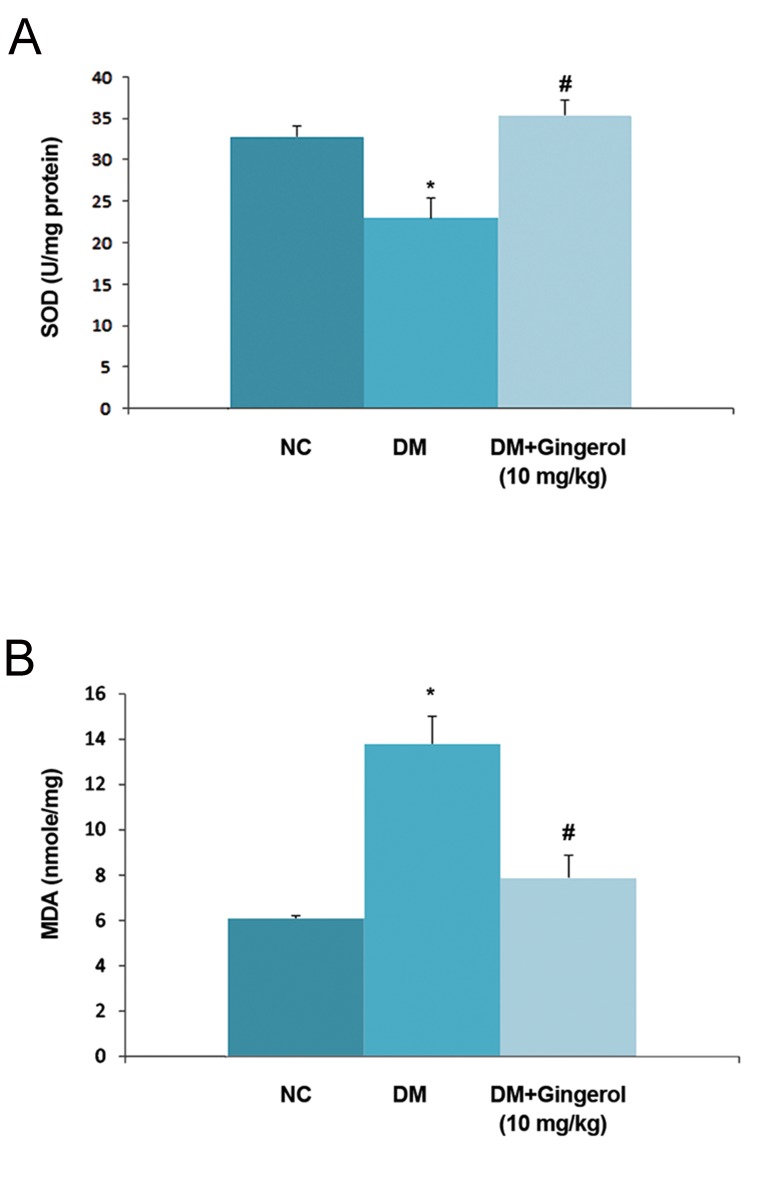
Effect of gingerol on the antioxidant status. A. Superoxide dismutase (SOD) and B. Malanaldehyde (MDA) levels for all of the rats. The data are presented as mean ± standard error of the mean. NC; Normal control, *; P<0.05 vs. control group and #; P<0.05 vs. diabetes mellitus (DM) group.

**Fig.7:**
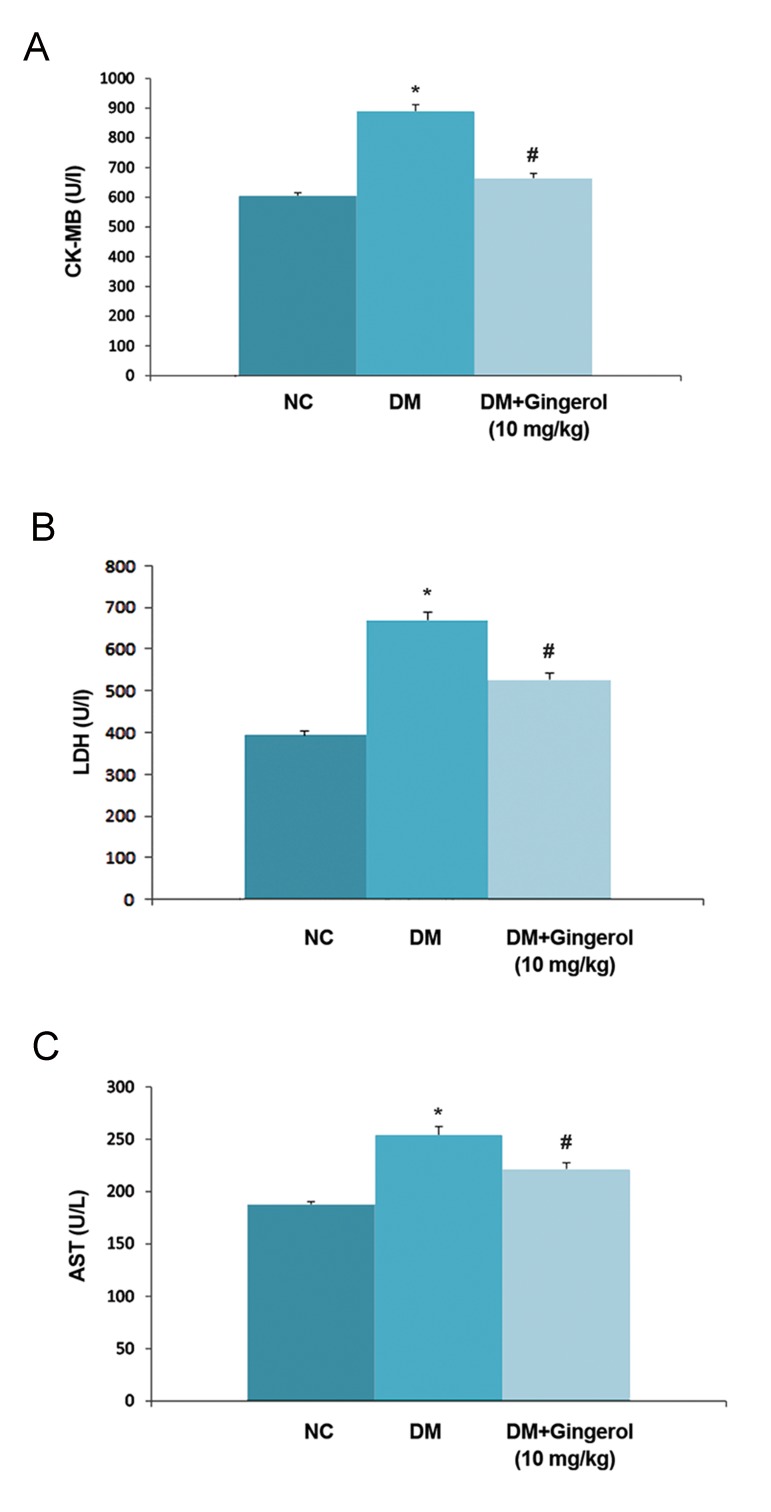
Effect of gingerol on the on the mycardial injury. A. Creatine kinase-MB (CK-MB), B. Lactate dehydrogenase (LDH), and C. Aspartate aminotransferase (AST) levels for all of the rats. The data presented as mean ± standard error of the mean. NC; Normal control, *; P<0.05 vs. control group and #; P<0.05 vs. diabetes mellitus (DM) group.

STZ induced DM rats showed significant (P<0.05) decline in SOD activity and enhanced malanaldehyde (MDA) activity which represented the accumulation of lipid peroxidase in the heart tissue compared with the NC group. There was significant (P<0.05) down-regulation of MDA activity and unregulated SOD activity in STZ-induced DM rats that received gingerol (Fig .6). The levels of LDH, AST, and CK-MB which are considered biochemical markers of myocardial damage (Fig .7). STZ-induced DM rats had significantly (P<0.05) enhanced levels of these three makers compared to the NC group. 

Subsequent treatment with gingerol in the STZ-induced DM rats showed that these myocardial enzymes significantly (P<0.05) decreased compared to the STZ-induced DM rats not treated with gingerol. 

### Gingerol inhibits streptozotocin-induced apoptosis of cardiomyocytes

**Fig.8:**
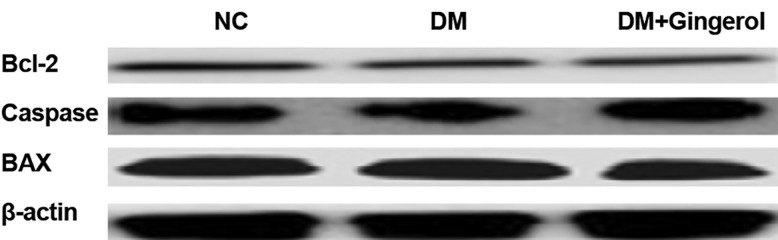
Effect of gingerol on the as determined by western blot analysis showing the protein expression levels of caspase 3, Bcl 2, and BAX. β actin was use d as the control. NC; Normal control and DM; Diabetes mellitus.

The inhibitory effect of gingerol against Bcl-2 (anti-apoptotic), caspase-3, and BAX (proapoptotic protein) were estimated via Western blot. STZ-induced DM rats had decreased Bcl-2 expression along with enhanced caspase-3 and BAX expressions compared with the NC group rats. The STZ-induced DM rats that received gingerol had significantly enhanced Bcl-2 expression and reduced expressions of BAX and caspase-3 compared to STZ-induced DM rats that did not receive gingerol (Fig .8).

## Discussion

In the current study, we observed marked increase in concentrations of serum and plasma glucose, which resulted from inhibition of insulin secretion induced via ROS caused by STZ. The increased ROS levels inhibited the antioxidant defense system and induced oxidative injury in the β-cells of the pancreas. Gingerol treated rats showed significant decline in the concentrations of serum and plasma glucose in STZ-induced DM rats. However, this decrease was not to the same level as the normal control group rats. The current study confirmed the decreased serum and plasma glucose levels. This confirmed reduced free radical generation and inhibited lipid peroxidation, even as guarding the β cells via enhancing the insulin secretion from the pancreatic β cells and obstructed oxidative stress induced via STZ (12, 13). STZinduced diabetic rats showed an effect on blood glucose and insulin levels due to abnormalities in β-cell functions (13). In the current study, administration of STZ confirmed the successful induction of DM via markedly enhanced serum glucose levels and elevated concentrations of TC and TG. In the current method, the serum symptoms showed that the enhanced levels of these markers resembled those of type 2 DM as compared with type 1 DM.

DCM is classified as ventricular dysfunction with elevated risk of cardiac failure, in the absence of coronary artery, valvular heart disease, or hypotension (14). The previously mentioned complications are regularly identified in animals and humans. The current study has shown that untreated DM rats had reduced or modulated antioxidant defense systems. This finding was confirmed by inhibition of SOD activity, accompanied by pro-survival pathway of Bcl-2 inactivation and increased production of myocardial lipid peroxidation, which ultimately ended cell apoptosis and enhanced the inflammatory reactions (15). The gingerol treated rats confirmed the preventive effect against the DCM via alterations of oxidative stress or inflammatory factors. The preventive effect of gingerol might be attributed to the reductions of the increased TG and blood glucose levels. 

Another mechanism of action of gingerol might be attributed to the attenuation of oxidative stress. The study results showed neutralization and minimization of ROS via its potent antioxidant defense system. Oxidative stress has shown the inequality among the generation and discharge of free radicals, which takes part in the progression of the heart disease and left ventricular remodeling in DCM (16,17). Hyperglycemia has been shown to aggravate glucose oxidation and production of mitochondria ROS, which further indicated the effect on DNA and increased apoptosis rate (18). In the current investigation, we observed that rats which received gingerol had reduced lipid peroxidation via decreased MDA levels and elevated activities of SOD enzymes in the STZ-induced DM rats. Inflammation plays a crucial role in diabetes. Our investigation has shown the reductions in cardiac inflammation characterized by elevated concentrations of inflammatory cytokines IL 6 and TNF α, which play a significant role in the manifestation of DCM (14,19). Gingerol is assumed to exert potential effects on different organs in DCM rats. 

## Conclusion

The current results suggested that the gingerol had greater therapeutic activity against DCM treatment and probably other cardiovascular diseases by modulation of inflammation, oxidative stress, metabolic abnormalities, and cellular apoptosis pathways.
